# Editorial: Neurogenic inflammation in paroxysmal disorders (migraine and epilepsy)

**DOI:** 10.3389/fneur.2023.1292357

**Published:** 2023-09-25

**Authors:** Zheman Xiao

**Affiliations:** Department of Neurology, Renmin Hospital of Wuhan University, Wuhan, Hubei, China

**Keywords:** migraine, epilepsy, inflammation, biomarkers, microglia

Epilepsy and migraine are among the most prevalent neurological disorders, characterized by recurrent episodes with an absence of symptoms (1). Neuroinflammation is thought to be an adaptive response caused by noxious stimuli such as infection, injury, and tissue stress, and plays an essential role in the pathogenesis of epilepsy and migraine (2, 3). In addition, inflammatory mediators could contribute diagnostic, prognostic, and predictive biomarkers for epilepsy and migraine, which would enable the stratification of patients in future clinical studies. This Research Topic aims to focus on the available evidence that biomarkers of inflammation have pathogenic value and could be therapeutic targets to explore immunomodulatory and anti-neuroinflammatory treatments for epilepsy and migraine. Six articles were published as part of the Research Topic covering different forms of epilepsy and migraine. Several studies have reported the association between inflammation and epilepsy. Wu Q. et al. discovered that downregulation of the TLR4/NF-κB inflammatory pathway in epilepsy could inhibit microglial activation and the expression of the inflammatory factor CD68, which could inhibit hyperphagocytosis, suppress epileptogenesis and exacerbation, and thus ameliorate cognitive and affective deficits after epileptic seizures. In addition, the study of Wu J. et al. aimed to investigate the genome-wide biological significance of the circulating miRNA markers found in peripheral whole blood of adult epileptic seizure patients by integrating analysis using bioinformatics approaches. Generalized convulsive epilepsy (GCE) is an important subtype of epilepsy. A bidirectional Mendelian randomized study by Wang et al. assessed the relationship between generalized convulsive epilepsy and systemic inflammatory modulators.

The levels of some migraine biomarkers differ between episodic migraine (EM) and chronic migraine (CM), but information on C-reactive protein (CRP) levels in EM and CM is conflicting. A case-control study of Park et al. revealed no change in interictal C-reactive protein levels in individuals with episodic and chronic migraine. In addition, the research of Qiu et al. showed 2-Deoxyglucose alleviates migraine-related behaviors by modulating microglial inflammatory factors in experimental model of migraine. Reducha et al. found complete Freund's adjuvant (CFA) surgically administered to the dura causes periorbital allodynia and increases CGRP positive fibers in the trigeminal ganglion. Further work is needed to investigate whether CFA administered to the dura could be used as a non-CGRP inflammatory migraine model.

In conclusion, the articles included in this Research Topic provided an overview on the recent neuroinflammation findings in paroxysmal disorders (migraine and epilepsy). In the context of migraine, neurogenic neuroinflammation refers to inflammatory responses in the central and peripheral parts of the trigeminal nervous system due to neuronal activity. Neurogenic neuroinflammation also plays a critical role in the pathogenesis of epilepsy. Seizures can produce activation of neuroinflammation, which in turn may be involved in the progression of epileptogenesis.

## Author contributions

ZX: Writing—review and editing.
